# Open Force Field BespokeFit: Automating Bespoke Torsion Parametrization at Scale

**DOI:** 10.1021/acs.jcim.2c01153

**Published:** 2022-11-09

**Authors:** Joshua T. Horton, Simon Boothroyd, Jeffrey Wagner, Joshua A. Mitchell, Trevor Gokey, David L. Dotson, Pavan Kumar Behara, Venkata Krishnan Ramaswamy, Mark Mackey, John D. Chodera, Jamshed Anwar, David L. Mobley, Daniel J. Cole

**Affiliations:** † School of Natural and Environmental Sciences, Newcastle University, Newcastle upon TyneNE1 7RU, United Kingdom; ‡ Boothroyd Scientific Consulting Ltd., 71-75 Shelton Street, LondonWC2H 9JQ, Greater London, United Kingdom; § The Open Force Field Initiative, Open Molecular Software Foundation, Davis, California95616, United States; ∥ Department of Chemistry, University of California, Irvine, California92697, United States; ⊥ Department of Pharmaceutical Sciences, University of California, Irvine, California92697, United States; # Cresset, New Cambridge House, Bassingbourn Road, LitlingtonSG8 0SS, Cambridgeshire, United Kingdom; ∇ Computational & Systems Biology Program, Sloan Kettering Institute, Memorial Sloan Kettering Cancer Center, New York, New York10065, United States; ° Department of Chemistry, Lancaster University, LancasterLA1 4YW, United Kingdom

## Abstract

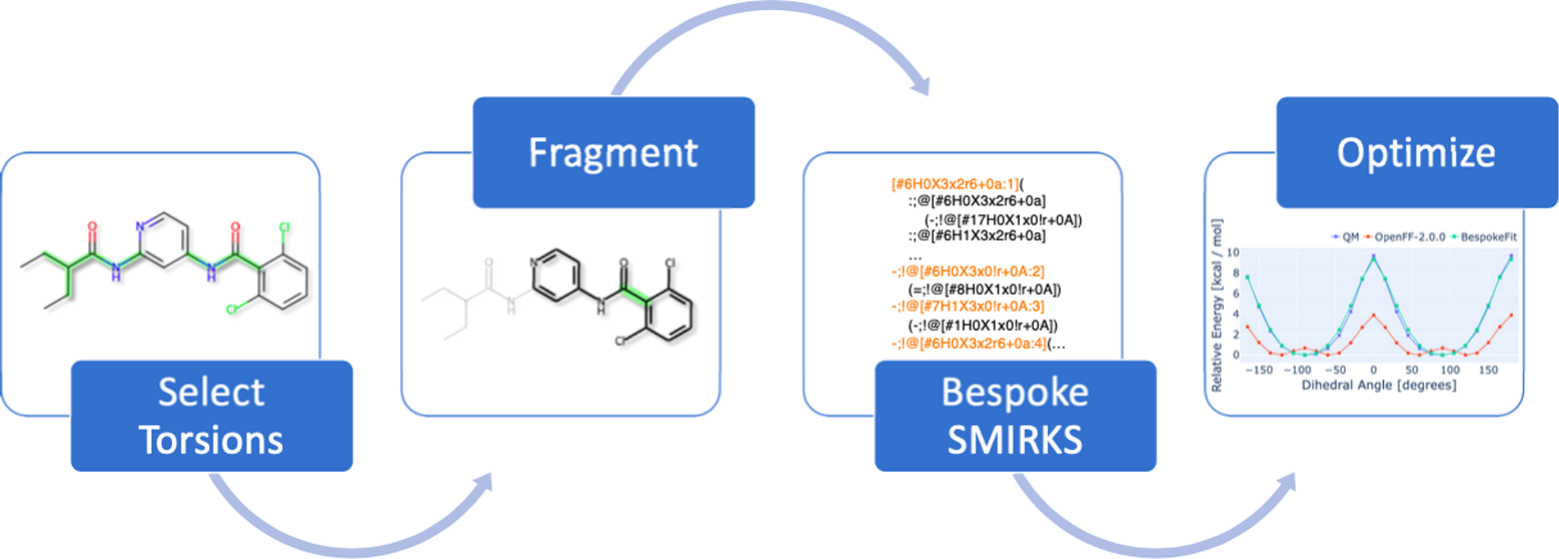

The development of accurate transferable force fields is key to realizing the full potential of atomistic modeling in the study of biological processes such as protein–ligand binding for drug discovery. State-of-the-art transferable force fields, such as those produced by the Open Force Field Initiative, use modern software engineering and automation techniques to yield accuracy improvements. However, force field torsion parameters, which must account for many stereoelectronic and steric effects, are considered to be less transferable than other force field parameters and are therefore often targets for bespoke parametrization. Here, we present the Open Force Field QCSubmit and BespokeFit software packages that, when combined, facilitate the fitting of torsion parameters to quantum mechanical reference data at scale. We demonstrate the use of QCSubmit for simplifying the process of creating and archiving large numbers of quantum chemical calculations, by generating a dataset of 671 torsion scans for druglike fragments. We use BespokeFit to derive individual torsion parameters for each of these molecules, thereby reducing the root-mean-square error in the potential energy surface from 1.1 kcal/mol, using the original transferable force field, to 0.4 kcal/mol using the bespoke version. Furthermore, we employ the bespoke force fields to compute the relative binding free energies of a congeneric series of inhibitors of the TYK2 protein, and demonstrate further improvements in accuracy, compared to the base force field (MUE reduced from 0.56_0.39_^0.77^ to 0.42_0.28_^0.59^ kcal/mol and *R*^2^ correlation improved from 0.72_0.35_^0.87^ to 0.93_0.84_^0.97^).

## 1 Introduction

The molecular mechanics force field (FF) is vital to the success of atomistic modeling of organic and biological systems. The FF encodes a library of transferable parameters that describe inter- and intramolecular interactions via physically motivated models defined by an atomic environment.^1−3^ These models offer users the ability to rapidly parametrize vast regions of small-molecule druglike chemical space and simulate the dynamics of complex, heterogeneous systems with low computational cost.

For FF-based molecular modeling to be worthwhile, the FF must be accurate. That is, it should accurately describe the potential energy surface of the target molecule and adequately describe the vital nonbonded interactions between the molecule and its (often condensed phase) environment. In an attempt to achieve this accuracy, most transferable FFs are parametrized following a similar philosophy. Specifically, a representative subset of small molecules is selected that contains key functional groups, such as those that appear frequently in druglike molecules.^1,4^ For these molecules, parameters are then fit to a combination of experimental and quantum mechanical (QM) data, and transferability between similar chemical environments is assumed.

Such an approach to transferable FF design is often successful as evidenced by numerous retrospective^7−10^ and prospective^11,12^ studies, which show good agreement between experiment and simulation. Critical applications of FFs include alchemical free energy calculations, which have become a widespread, relatively low-cost computational tool to aid the identification and development of high-binding-affinity small molecules in the early stages of drug discovery campaigns.^11^ However, due to the vast size of chemical space, and the local limitation of atom types used to describe these environments, the number of parameters required for broad, accurate coverage has tended to increase dramatically during FF development. For example, the most recent OPLS3e FF library contains ∼150 K torsional parameters (Table 1).^4^

**Table 1:** Number of Valence Parameters in a Selection of Modern Force Fields

parameter type	MMFF^5^	OPLS3^5^	OPLS3e^4^	Sage (OpenFF 2.0.0)^6^
bond stretching	456	1187	1187	88
angle bending	2283	15,236	15,235	40
torsional rotation	520	48,142	146,669	167

In an attempt to counter this trend, in a new line of general FFs, the Open Force Field (OpenFF) Initiative has replaced atom-typed parameter encodings with a technique termed direct chemical perception.^10,13^ The chemical perception framework assigns parameters via standard chemical substructure queries implemented in the SMARTS language. This removes many redundancies, for example, in equivalent parameters that would otherwise be applied to different combinations of atom types, and allows the OpenFF line of FFs (Parsley,^10^ Sage,^6^ etc.) to be very compact without sacrificing accuracy (Table 1). Given the hierarchical nature of these FFs, their extension becomes trivial. More specific substructure queries can be introduced for problematic areas of chemistry without affecting the more general, transferable parameters.

**Figure 1:**
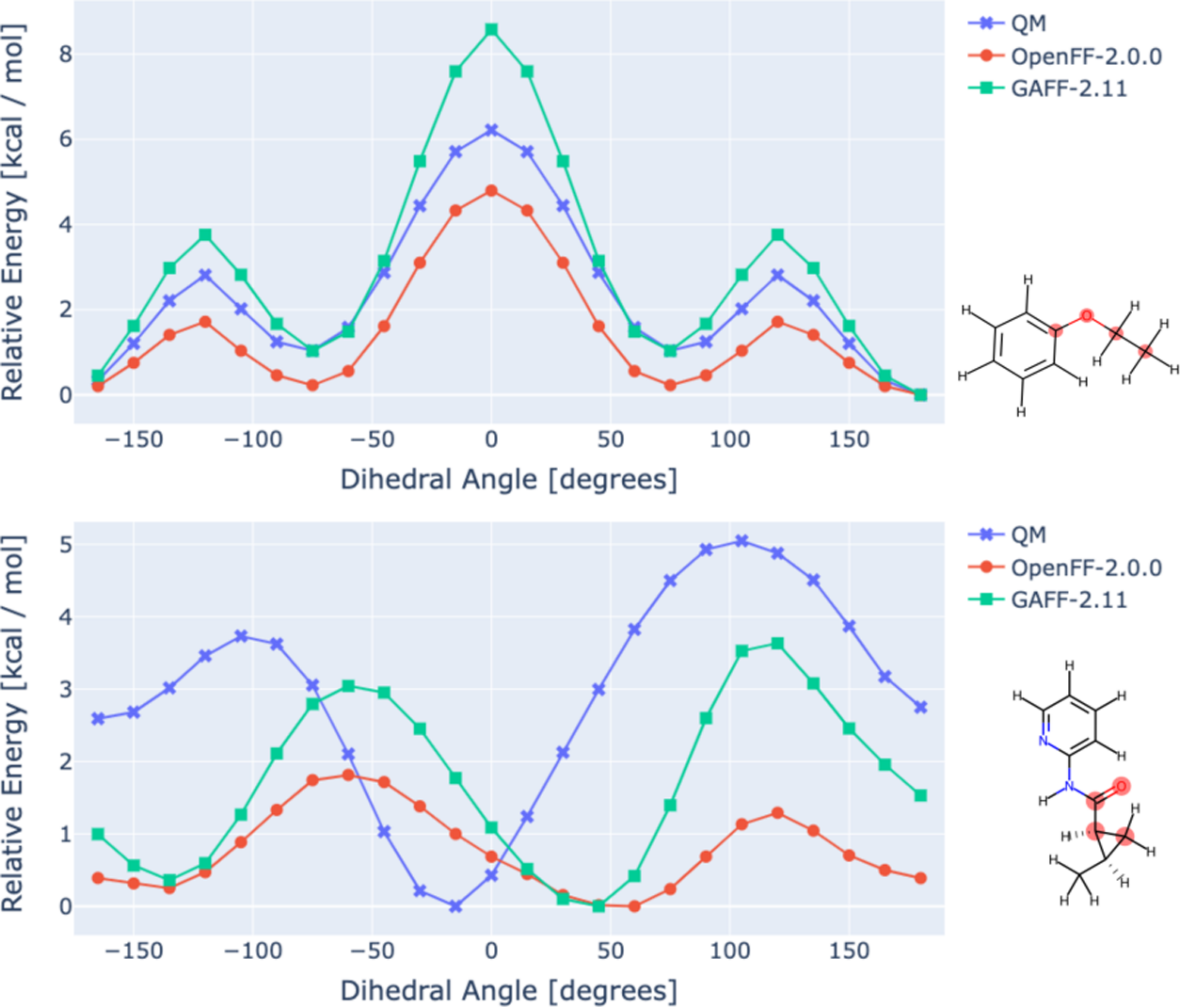
Ability of a force field to accurately reproduce the torsional potential energy surface depends on the complexity of the local chemical environment of the molecule. (Top) Example of good agreement between potential energy surfaces generated using OpenFF 2.0.0 and GAFF 2.11 parameters, compared to QM, for a simple molecule. (Bottom) More complex druglike fragment where reparametrization is necessary. The OpenFF and GAFF implementations used here both assigned partial charges using AM1-BCC.

The OpenFF family of FFs has been shown to offer competitive accuracy when benchmarked against QM geometric and energetic properties^14^ despite having significantly fewer parameters. However, torsion parameters, in particular, are known to be sensitive to the local environment within the target molecule and may be expected to be less transferable than the other valence parameters. Torsional parameters must account for many stereoelectronic and steric effects.^15^ In addition, resonance effects between aromatic rings, for example, can mean that even nonlocal substitutions, which may not be captured via chemical perception, can affect torsional profiles.^16^Figure 1 compares example potential energy surfaces of two molecular fragments calculated with contemporary general force fields OpenFF 2.0.0 (Sage) and GAFF 2.11, with a QM reference (see Section 2). While the default, transferable torsional parameters show good performance in some cases (top panel), more complex chemical environments can lead to an inaccurate reproduction of the QM potential energy surface (lower panel), resulting from poor transferability. Thus, due to the complexity encoded in torsional parameters, and the resulting poor or partial transferability, they are often the target for reparametrization. To this end, several automated methods exist to derive torsion parameters that are specific to the target molecule under study. For example, an automated torsion parametrization package, named FFBuilder supplements the already extensive set of base library parameters in the proprietary OPLS3 FF.^4^ This allows users to fit new torsion parameters for novel chemistry that is poorly represented by the general FF using a consistent parametrization method. Several other tools also aid the fitting of bespoke torsion parameters to QM potential energy surfaces; these include QUBEKit,^17,18^ paramol,^19^ parmfit,^20^ qforce,^21^ JOYCE,^22^ DFFR,^23^ Rotational Profiler,^24^ or the algebraic method of Kania,^25^ to name a few. Although bespoke torsion parameters have the potential to improve the accuracy of molecular simulations, fitting these parameters to multiple QM torsion scans can significantly slow down the parameter assignment stage for users. However, there is now the opportunity to make use of recent advances in machine learning (e.g., ANI^26,27^)- and semiempirical (e.g., xTB^28^)-based approaches, which are in general intermediate in accuracy and computational expense between MM and full QM. While these approaches are currently too slow for routine molecular dynamics sampling in the condensed phase, their use for the rapid generation of reference data for parametrizing FFs is appealing, as demonstrated by the recent refinement of GAFF-2 parameters against ANI2x torsional scans.^29^

Here, we present OpenFF BespokeFit, an open-source, automated python package for bespoke FF parameter fitting. This first version of BespokeFit specifically aims to derive bespoke torsion parameters for individual molecules but we plan to extend it to additional FF terms in future. BespokeFit is designed for compatibility with the SMIRKS Native Open Force Field (SMIRNOFF) format; hence, it uniquely provides users with the opportunity to re-fit torsion parameters, using robust methods that are consistent with the base OpenFF parametrization philosophy. We make use of the unified quantum chemistry (QC) program executor QCEngine^30^ to provide simple, resource-agnostic access to a wide range of quantum, semiempirical, and machine learning-based reference data, which can be generated on-the-fly. Furthermore, we introduce OpenFF QCSubmit as an open-source tool for curating, submitting, and retrieving large QM reference datasets from QCArchive^31^ to aid large-scale FF parameter fitting. We demonstrate the utility and ease of use of the BespokeFit/QCSubmit interface by deriving bespoke torsion parameters for a large dataset of 671 QM torsion scans derived from fragments of druglike molecules. We further demonstrate the ability of BespokeFit to construct FFs for a congeneric series of inhibitors of the TYK2 protein, and benchmark the accuracy of the resulting FFs by computing protein–ligand binding free energies and comparing against experimental data.

## 2 Methods

### 2.1 BespokeFit Design

OpenFF BespokeFit is a scalable and extensible framework that automates the optimization of bespoke torsion parameters for SMIRNOFF-style FFs against QC reference data. It is designed with reproducibility and ease of use in mind. The process begins by defining a (or retrieving the default) workflow protocol that defines the entire fitting process, which typically involves four stages: (1) fragmentation, (2) SMIRKS generation, (3) QC reference data generation, and (4) parameter optimization. This general workflow can then be applied to a target set of input molecules, producing a series of specific fitting schemas that can be submitted to BespokeFit for processing. The general workflow can also be serialized to file in JSON format for later use and shared with others to ensure reproducibility of the fitting protocol.

The default workflow protocol offers an automated means to extend the base OpenFF parameters directly from the command line with established and robust protocols that are consistent with the optimization procedure used for the original FF. For example, the following command could be used to parametrize acetaminophen:

openff-bespoke executor run --smiles “CC(=O)NC1=CC=C(C=C1)O”

--output “acetaminophen.json” --output-force-field “acetaminophen.offxml”

The modular design of the code base makes building the workflow straightforward, as users can simply select the module they wish to use for each stage of the workflow and add it to the schema. For example, users can choose between two predefined fragmentation modules, which offer rule- or heuristic-based fragmentation.^16^ Extending the workflow is also trivial as users can add new modules for any of the above fitting stages using the plugin framework. This allows for fast prototyping of new modules, such as the addition of new parameter optimization methods, without needing to update the core package, which is critical in keeping up with the developing landscape of QC-based parameter derivation. Next, we discuss the various stages of a typical BespokeFit workflow.

### 2.2 Fragmentation

Torsion-preserving fragmentation can significantly speed up the generation of reference QM torsion scans while providing a close surrogate potential energy surface of the associated torsion in the parent molecule. BespokeFit uses the OpenFF Fragmenter^16^ package to fragment larger molecules into smaller representative entities. As well as reducing the number of degrees of freedom to minimize at the QM level during a torsion scan, fragmentation can also help to avoid hysteresis by reducing the opportunities for steric clashes in more complex molecules.

**Figure 2:**
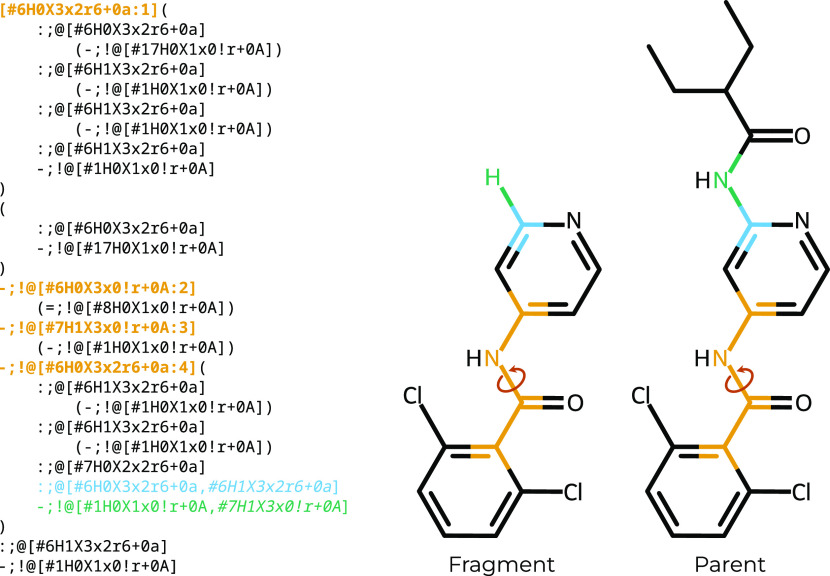
BespokeFit uses SMIRKS patterns, which include the maximum common substructure between the fragment and parent molecule, to assign bespoke torsion parameters. (Right) Parent and corresponding fragment produced via WBO fragmentation around the rotatable bond highlighted with a circular arrow. (Left) BespokeFit SMIRKS pattern corresponding to this dihedral angle, with the four tagged atoms highlighted in yellow. The blue and green highlighted text in the pattern indicate 'or' statements, which account for differences between the parent and fragment molecules.

Fragments are constructed around each nonterminal rotatable bond (that is, any bond that is not triple-bonded, is not in a ring, or does not include an atom with a valence of one). An example is shown in Figure 2. Fragmenter aims to preserve the local environment around the targeted torsion while retaining as little of the parent molecule as possible to reduce the computational demands of the calculation. However, oversimplification of the local chemical environment can result in fragments that inaccurately approximate the parent potential energy surface, which may lead to parameters that transfer poorly back to the parent molecule. It has been shown that the Wiberg bond order (WBO) provides a fast and robust measure of whether a torsion profile has been disrupted by fragmentation.^16^ BespokeFit then only accepts a proposed fragmentation if the WBO of the parent and proposed fragment agree to within a defined threshold (0.03 e by default).

### 2.3 SMIRKS Generation

Having generated the fragments for parametrization, the dihedral angle to be scanned must be assigned a SMIRKS Native Open Force Field (SMIRNOFF) format parameter for incorporation into the FF. When adding any new parameter to a force field, a trade-off between accuracy and transferability must generally be made to avoid the proliferation of parameters and/or atom types. However, in the case of bespoke parametrization, accuracy is favored, and so highly specific encodings are used to ensure the patterns can only be reused in similar chemical environments, rather than being applied to chemistries that they were not intended to cover. Each fragment is considered to be the minimum electronically decoupled substructure that preserves the local chemical environment of the torsion (due to the conservation of the WBO). Hence, the maximum common substructure between the parent and fragment molecules is embedded into the SMIRKS pattern used to label the scanned dihedral. This SMIRKS pattern is produced by the ChemPer package,^32^ and is linked to the generated bespoke torsion parameters, thus facilitating transferability between the fragment and the parent, as well as any other molecules that share this exact substructure (common in studies of congeneric series, as we shall show later). Figure 2 shows an example of a molecule, and the corresponding fragment, alongside the SMIRKS pattern generated for the highlighted torsion scan. The reader does not need to be too familiar with SMIRKS patterns to recognize that every possible attribute has been added to the pattern, making it highly specific to the molecule under study.

**Figure 3:**
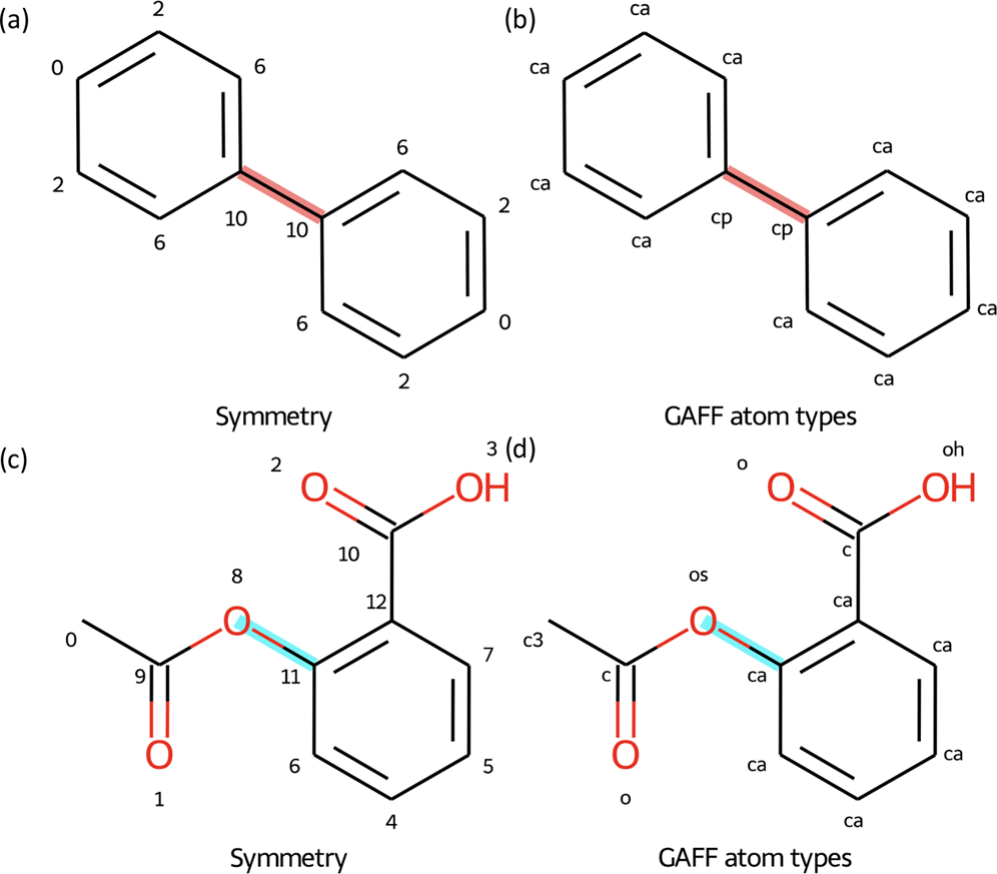
BespokeFit uses the symmetry defined by the bonding topology of the molecule to group torsion parameters. Grouping like torsions using the symmetry defined by the bonding environment of the molecule can recover GAFF torsion types in simple cases. (a) Biphenyl is shown with the symmetry labels as determined by RDKit for each nonhydrogen atom or (b) the atom types assigned via GAFF. Using symmetry labels to identify torsions through the highlighted central rotatable bond produces one unique torsion type (6–10–10–6) consistent with the unique combinations of GAFF atom types (ca–cp–cp–ca). (c) Aspirin is shown with the symmetry labels or (d) GAFF atom types. Using symmetry labels to identify torsions through the highlighted (8–11)/(os–ca) bond produces two unique types (9–8–11–6 and 9–8–11–12), whereas the combination of GAFF atom types leads to a single torsion type (c–os–ca–ca).

An additional decision to make is how many parameters should be used to parametrize a given torsional rotation? Rotatable bonds can have many torsion parameters associated with them, due to the number of unique combinations of atom quartets running through the central bond, and the overall torsional profile is given by the sum of these terms. Using a traditional atom-typed parameter assignment scheme can lead to insufficient flexibility. For example, for the highlighted rotation in aspirin, shown in Figure 3d, atom-typed schemes would use a single set of torsion parameters, corresponding to the GAFF atom types c–os–ca–ca.

Instead, BespokeFit groups the torsion parameters using symmetry labels as defined by the bonding topology of the molecule. In the case of the highlighted rotatable bond in aspirin, two unique torsions (9–8–11–6 and 9–8–11–12) are identified due to the terminal atoms (labeled 6 and 12) having different local environments (Figure 3c). As SMIRKS patterns can be more expressive than the combination of predefined atom types, we can create a single bespoke pattern that matches all of the torsions with identical symmetry labels (as determined by RDKit^33^ or the OpenEye toolkit^34^). This allows BespokeFit to automatically introduce extra parameter flexibility via the splitting of torsion parameters into unique symmetry types, which has been shown to be essential for accurate reproduction of reference potential energy surfaces.^19^ Conversely, a similar analysis is also shown in Figure 3a,b for biphenyl, where symmetry labels avoid the unnecessary splitting into separate torsion types and simplify the parameter optimization step. Thus, the use of symmetry labels and SMIRKS patterns in BespokeFit provides an automated means to optimally select torsion types for fitting.

Finally, once the torsion parameters have been fit, the parameters are added back into the main FF library. Traditionally, in atom-typed FFs, introducing a new torsion parameter would require the creation of new, potentially redundant atom types. However, since OpenFF uses separate substructure searches for each parameter type (along with a separate parameter hierarchy), the introduction of new torsion parameters does not complicate other parts of the FF, nor does it create issues with conflicting or complex atom types. Instead, the new parameter is simply placed at the bottom of the hierarchy to ensure that it takes precedence when the exact substructure is identified in future parameter assignment workflows.^13^

### 2.4 Reference Data Generation

A vital component of the BespokeFit workflow is the reliable generation of accurate reference (for example, QM, semiempirical, or machine learning potential) data against which to optimize the force field parameters. BespokeFit interfaces with the TorsionDrive^35^ package to automatically perform one-dimensional (1D) torsion scans around the targeted rotatable bond in each fragment molecule. Consistent with the methods used to parametrize the underlying FFs, TorsionDrive schedules a series of geometry optimizations via the geomeTRIC package,^36^ with the targeted dihedral angle constrained to values drawn from a regularly spaced grid (from −180 to 180°). TorsionDrive makes use of wavefront propagation to re-seed neighboring grid points with new low-energy structures, which helps to avoid hysteresis in scans where multiple rotatable bonds are present.^35^

To offer flexibility in the generation of the reference data, BespokeFit makes extensive use of QCEngine,^30^ which is a program executor and IO standardizer, offering one unified interface to a plethora of QM, ML, MM, and semiempirical computation backends. Due to this unified interface and the plugin nature of QCEngine, new computational reference methods can be rapidly prototyped and used with BespokeFit, with no changes to the source code. For example, as more accurate and faster ML potentials become available in QCEngine, it will be trivial to make them available in BespokeFit for next-generation FF fitting. Furthermore, due to the standardization of the output from QCEngine, BespokeFit is also able to use preexisting QC reference data. These data may be held in private repositories, for example, or obtained from the public MolSSI QCArchive project, which is a platform for computing, organizing, and sharing QC data.^31^ The advantages of these interfaces to QCEngine and the public QCArchive project will be explored further in Case Study 1. Full information concerning the reference data generated in this study is provided in Supporting Information Section S1.1.

### 2.5 QCSubmit Design

The development and benchmarking of the valence terms in modern high-quality FFs can require execution and collection of thousands of QM calculations,^14^ often conducted via complex error-prone workflows involving multiple scripts, file formats, and software with little to no provenance. With increased computational power via modern hyper-threaded CPUs or HPCs, FF developers have the ability to generate QC data at an unprecedented scale. However, it is prohibitively time-consuming to manually build datasets of the size that will be required in BespokeFit, for example. Distributed compute and database platforms, such as QCFractal,^31^ were designed to overcome these issues and make the orchestration and storage of large-scale QM calculations trivial. However, the community is currently lacking robust tools to assist in managing the creation, submission, and collection of large datasets, and handling their interaction with public or private QCFractal instances.

Here, we present OpenFF QCSubmit as an open-source framework to curate and schedule large QC datasets, and retrieve them from any QCFractal instance, including the public QCArchive. In particular, the framework aims to define reproducible workflows for the construction of QC datasets with a range of purposes, including but not limited to single-point Hessian calculations, global optimizations, and torsion drives. QCSubmit also provides an extensive set of modular workflow components that can be combined in any order to produce unique dataset creation pipelines starting from large collections of input molecules. These components enable common preprocessing operations such as filtering, state enumeration, fragmentation, and conformer generation. Figure S1 shows an example of how a “TorsiondriveDatasetFactory”, of the type required for BespokeFit input, can be constructed using a selection of configurable workflow components in a python script.

**Figure 4:**
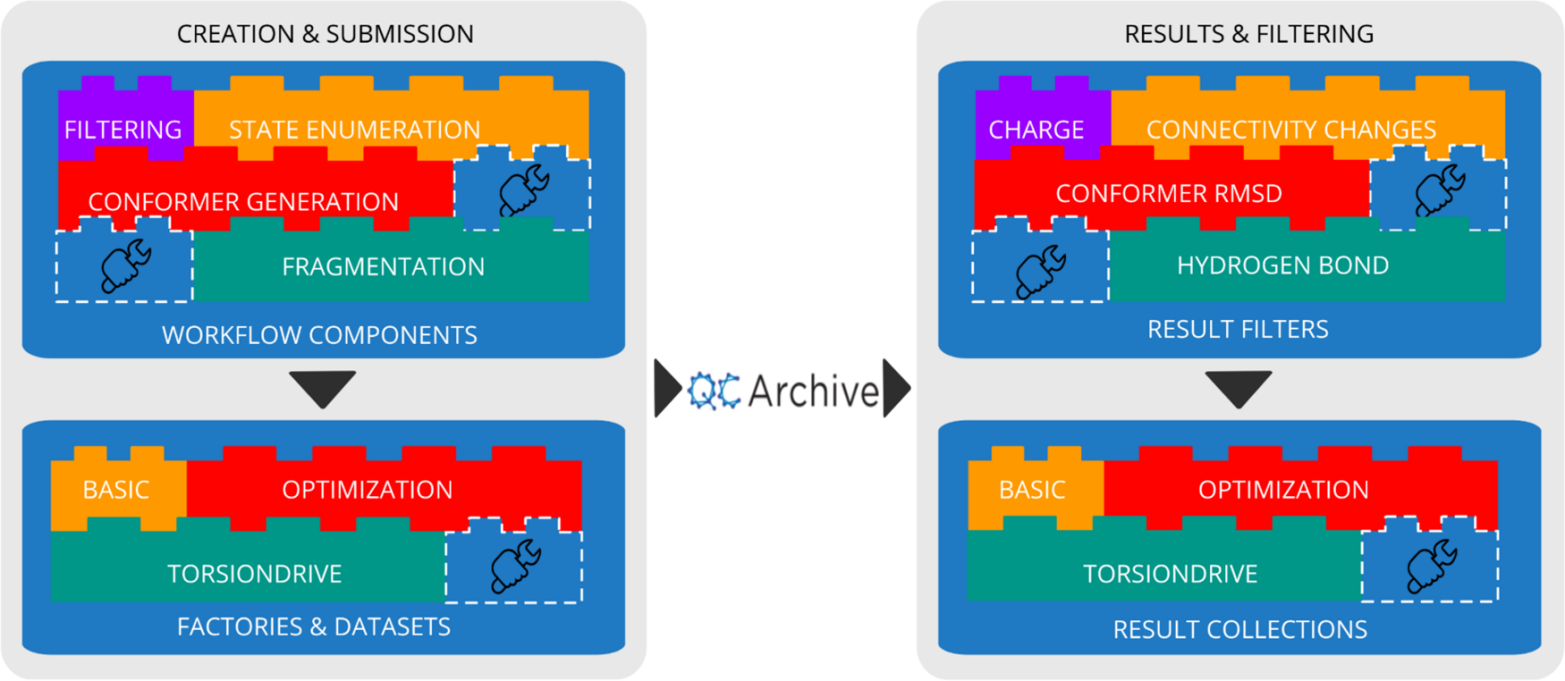
Overview of the QCSubmit package and its modules, where dashed blocks represent the plugin nature of the framework. Here, QCArchive serves as a distributed computing platform to coordinate and store large datasets of QC calculations. QCSubmit wraps around QCArchive, and other QCFractal instances, to streamline the creation and submission of large datasets as well as the retrieval and filtering of results.

QCSubmit also facilitates the aggregation of completed calculations across multiple QCFractal instances and datasets into a single local results collection. In line with the dataset creation workflow, results collections can also be processed with many common filtering components such as net charge, element coverage, or SMARTS queries, which allows users to construct customized datasets for training or testing of FFs, or even ML models. Datasets can be serialized to JSON and used as a source of provenance. These are lightweight references to the calculations that store only vital information, such as the SMILES,^37^ InChlKey,^38^ and QCFractal record identification, but can also be used to quickly pull down the associated records to access the raw data. This enables users to do quick local filtering for specific molecule data, including proprietary molecules, without contacting the QCFractal instance until the raw record data is required. Figure 4 gives a broad overview of the modular plugin-based architecture of QCSubmit, which enables efficient large-scale communication with private or public QCFractal instances.

### 2.6 Parameter Optimization

The BespokeFit workflow concludes with the optimization of the torsion parameters to the QM (or alternative) reference data using an interface with the ForceBalance package.^39^ ForceBalance iteratively optimizes the FF parameters related to the torsional potential, which in the case of the class 1 additive FF employed here, is described by the following truncated Fourier series
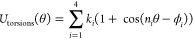
1where *k*_*i*_ are the torsion force constants, *n*_*i*_ are the periodicities, and ϕ_*i*_ are the phases of the torsion potentials. During the optimization, we hold the periodicities, phases, and 1–4 scaling factors fixed, and only optimize the *k*_*i*_ parameters. By default, the starting parameters (phases, periodicities, and force constants) for each bespoke torsion are assigned using the base OpenFF FF. Extra degrees of freedom are then introduced by fully expanding the periodicities of the torsion term to include all integers from *n*_*i*_ = 1 to 4, and an initial value of zero is given to the associated *k*_*i*_ parameters of newly introduced terms.

ForceBalance is then used to optimize the set of FF parameters (Φ) via least-squares minimization of an objective function comprising a weighted sum of the squared deviation between the QM and MM potential energy surfaces across all targeted torsion angles
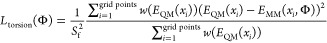
2where *S*_*f*_ = 1.0 kcal/mol is a scaling factor, and *E*_QM_(*x*_*i*_) and *E*_MM_(*x*_*i*_) represent the relative energies of conformations *x*_*i*_ compared to the QM and MM global minima, respectively. A weight factor *w*(*E*) controls the contribution of each grid point to the objective function. It is constant up to a first cutoff (1.0 kcal/mol) and then attenuates to the second hard cutoff (10.0 kcal/mol) after which all weights are zero:
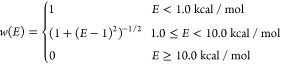
3

Following the methods used to parametrize the base OpenFF, at each grid point, the four atoms forming the targeted dihedral are held fixed, and all remaining atoms undergo a MM relaxation with a positional harmonic energy restraint of 1 kcal/mol/Å^2^. This ensures that the overall conformation of the molecule remains close to the QM minimum, while ensuring that the torsion parameters do not have to compensate for deficiencies in the other terms in the FF, such as overly stiff repulsive LJ interactions with nearby atoms in the molecule.

All bespoke torsion parameters across the multiple fragments that make up the target molecule are then optimized simultaneously, which helps to capture any coupling between connected torsions. The total objective function of the optimization including a parameter regularization penalty is then given by
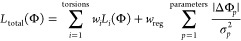
4The L1 regularization penalizes the absolute difference between the optimized torsion parameters and their initialized values, |ΔΦ_*p*_|, and can effectively remove the contribution of redundant parameters by shrinking their coefficients to zero. As BespokeFit makes no assumption a priori on the expected periodicities or number of unique torsion parameters required for an accurate fit, this can lead to a high number of optimizable parameters. Thus, L1 regularization is made the default, though L2 regularization is also available in ForceBalance if desired. Usually, the regularization term prohibits large deviations from the initial parameters. However, over-fitting is less of a concern during bespoke parametrization, and so the default prior widths (σ_*p*_) on the parameter restraints are increased (from 1.0 to 6.0) so as to not limit the maximum achievable accuracy of the optimization due to poor initial guesses.

Once the parameter optimization has converged, the final bespoke parameters can be cached locally into BespokeFit and can be reused. In the case of a congeneric series, this can save considerable time if BespokeFit determines that parameters for a general core can be reused, that is if Fragmenter generates the exact same fragment for a given torsion scan.

## 3 Results

### 3.1 Case Study 1: Large-Scale QC Data Generation and Bespoke Parameter Optimization

To demonstrate the utility of BespokeFit in deriving accurate, bespoke torsion parameters at scale, we have chosen to parametrize the entire dataset of ligands compiled by Wang et al.^40^ The dataset comprises 199 druglike molecules taken from eight congeneric series, with diverse chemical moieties and a range of net charges. The dataset is often used to validate FF accuracy in the context of free energy calculations,^10,40−42^ and a possible avenue to accuracy improvements is through the bespoke parametrization of the torsion parameters. Here, we will show that the accuracy of the MM potential energy surface about each torsion angle can be substantially improved via bespoke parametrization, compared to the base FF.

First, to distribute the required QC calculations across multiple HPCs worldwide and store the calculations for public use, we created an OpenFF QCSubmit workflow to process the molecules and create torsion drive datasets compatible with QCFractal. The workflow processed the multiple ligand SDF files as input and fragmented the molecules using the WBO fragmentation workflow component with default settings. Up to four diverse conformers for each fragment were produced, using OMEGA from OpenEye^43^ to seed the torsion scans. The workflow resulted in a torsion drive dataset comprising 490 molecular fragments and 671 unique scans. OpenFF QCSubmit was then used to submit the dataset to the public QCArchive instance with two different compute specifications, the default OpenFF QC method (B3LYP-D3BJ/DZVP^44−47^), using the PSI4 package,^48^ and the GFN2-xTB semiempirical method^28^ (Supporting Information Section S1.1). Once all of the calculations were complete, a local BespokeFit server was set up to generate and optimize bespoke torsion parameters. Each fragment was initially parametrized using the base OpenFF 2.0.0 (Sage), and then torsion parameters were optimized against the chosen reference data (that is, either the default QC or the xTB torsion scans), following the procedure described in Section 2.6.

Once the parameter optimization was complete, we analyzed the accuracy of the new parameters by computing deviations between QM and MM geometries and energy profiles. In particular, starting from the QM optimized geometry for each fragment at each point on the torsion scan, the conformer underwent a full MM relaxation, with only the targeted torsion angle being fixed. The MM energy was recorded at the final relaxed geometry and the root-mean-square error (RMSE) was computed over the full scan, relative to the QC reference relative energies. The root-mean-square deviation (RMSD) was also computed between QM and MM relaxed coordinates, and the maximum value across the scan was recorded.

**Figure 5:**
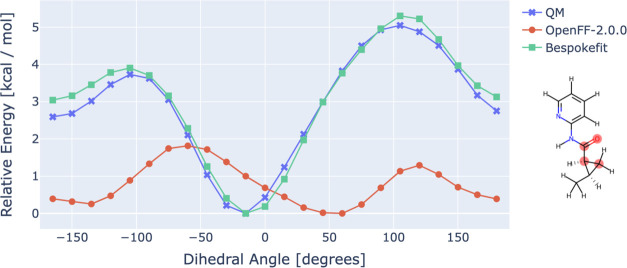
Bespoke dihedral parameters improve the accuracy of the base force field. Example of force field performance for a fragmented molecule from the Wang dataset,^40^ using OpenFF 2.0.0 and the same FF augmented with bespoke torsion parameters (see also Figure 1).

The average RMSE in the energy profiles and RMSD in the relaxed coordinates, for both the base Sage and bespoke FFs, relative to the QC torsion scans are shown in Table 2. As expected, the bespoke parametrization shows a clear improvement in the energy profiles across the set of 671 torsion scans, with the average RMSE reducing from 1.1 (base Sage) to 0.4 kcal/mol (Sage+BespokeFit). Figure 5 exemplifies the improvement in the potential energy surface using BespokeFit, compared with the QM reference data and base Sage parametrization. It is important to emphasize that we would not expect the energy error using BespokeFit to reach zero, both because of limitations in the FF functional form, and because the default BespokeFit workflow attenuates the contributions of any reference data with relative energy between 1.0–10.0 kcal/mol above the minimum (see Section 2.6). A further 12 representative torsion scans are shown in Figures S2–S4 and demonstrate a generally very good agreement between BespokeFit and QM torsion scans at low energies, with some larger deviations in the high-energy regions.

**Table 2:** Performance of Sage and BespokeFit Parameters on the Fragmented Wang Dataset,^40^ Using the Default QC Chemistry as the Reference Method^a^

force field	Max RMSD (Å)	RMSE (kcal/mol)
Sage (OpenFF 2.0.0)	0.652_0.610_^0.696^	1.096_1.051_^1.144^
Sage+BespokeFit	0.614_0.571_^0.658^	0.419_0.393_^0.449^
Sage+BespokeFit (no restraints)	0.748_0.510_^1.045^	0.351_0.262_^0.458^
Sage+BespokeFit (no restraints, RMSD)	0.574_0.533_^0.617^	0.349_0.327_^0.372^

a The final two rows test details of the parameter optimization procedure (with differences in procedure noted in parentheses), as described in the main text.

Finally, Table 2 demonstrates that similar to the base Sage FF, the final relaxed geometries using the Sage+BespokeFit parametrization remain close to the QC relaxed structures after full optimization with all restraints relaxed (with maximum RMSDs around 0.6–0.7 Å). The relaxed MM coordinates are also affected by the other valence and nonbonded terms in the force field, and so again perfect agreement with QM is not expected.

For completeness, the final two rows of Table 2 investigate the effects of removing the weak (1 kcal/mol/Å^2^) restraints on the atoms during the MM optimization stage of the fitting procedure (see Section 2.6). Removing all restraints (Sage+BespokeFit (no restraints)) slightly improves the fit to the QM potential energy surface (0.35 kcal/mol), as might be expected. However, this is at the expense of increasing the distances between QM and MM optimized structures (maximum RMSD between optimized structures increases to 0.75 Å). An alternative scheme that we have implemented allows us to add the RMSD between QM and MM optimized structures directly into the ForceBalance objective function (Supporting Information Section S1.2). This removes the need for weak restraints and adds small improvements in both energetic (RMSE reduced to 0.35 kcal/mol) and geometric (RMSD reduced to 0.57 Å) measures of agreement with QM. For consistency with the base FF, however, we retain the Sage+BespokeFit method, with weak restraints, as the default behavior in BespokeFit.

### 3.2 Case Study 2: Congeneric Series Optimization for Free Energy Calculations

As we have shown in Case Study 1, the introduction of data generation, curation, and sharing tools (QCFractal, QCArchive, QCSubmit), in combination with automated parametrization workflows (BespokeFit), open the possibility of the routine use of bespoke parameter derivation in applications such as alchemical free energy calculations. To validate the use of OpenFF BespokeFit in such drug discovery efforts, we compute here relative binding free energies for a congeneric series of inhibitors of the TYK2 protein parametrized with BespokeFit-derived FFs. Furthermore, to highlight the flexibility of the reference data generation via QCEngine, we derive FFs for the ligands from multiple sources, including the xTB semiempirical method and QC at the OpenFF default specification level (Supporting Information Section S1.1).

The target system is part of the Wang benchmark series^40^ discussed in Case Study 1, and so the fragments of each of the 16 ligands have already been processed and reference scans are available in QCArchive.^31^ Once again, a BespokeFit optimization server was set up locally and the QCArchive torsion scans were downloaded using the cache update CLI tool. Each ligand was initially parametrized using OpenFF Parsley 1.3.0 and was further processed using the default BespokeFit parametrization workflow as outlined in Section 2.

The full set of ligands, with the common core identified by Fragmenter highlighted, is shown in Figure S5. Due to the congeneric nature of the series, it is easy to see that there is a common core shared between the TYK2 ligands used in this study, and this core is also identified by the fragmentation procedure as being electronically decoupled from the proposed substitutions. To save parametrization time, the internal parameter caching system of BespokeFit was used to process a single molecule (id ejm-31) to generate a set of shared, core parameters. For the remaining molecules, bespoke parameters were only derived for new substitutions, in the presence of the optimized common core parameters. This reduces the parameter optimization time as the three rotatable bonds of the central core, and their corresponding 32 free parameters, do not need to be reoptimized for each of the 16 ligands in the series.

Table 3 reports the accuracy of a range of force fields, relative to the default OpenFF QC reference method, specifically for the TYK2 set, using the same metrics as in Case Study 1. As shown in the first row, the base OpenFF (1.3.0 in this case) has similar accuracy to that shown earlier, with energetic errors > 1 kcal/mol. Again, using the default BespokeFit workflow and fitting directly to QM scans (final row), we see improvements in both geometric and energetic (RMSE < 0.3 kcal/mol) measures of the force field accuracy.

**Table 3:** Performance of Parsley and BespokeFit Parameters on the TYK2 Dataset, Relative to the Default QC Scans^a^

force field	Max RMSD (Å)	RMSE (kcal/mol)
OpenFF 1.3.0	0.561_0.435_^0.698^	1.097_0.89_^1.328^
BespokeFit (GFN2-xTB)	0.375_0.28_^0.487^	0.792_0.701_^0.896^
BespokeFit (ANI2x//GFN2-xTB)	0.344_0.259_^0.442^	0.744_0.635_^0.875^
BespokeFit (B3LYP-D3BJ/DZVP//GFN2-xTB)	0.330_0.273_^0.388^	0.604_0.530_^0.697^
BespokeFit (B3LYP-D3BJ/DZVP)	0.311_0.251_^0.378^	0.289_0.235_^0.352^

a Methods in parentheses indicate the reference data used for fitting, where the notation “*x*//*y*” indicates that single-point calculations were performed with method *x*, using geometries optimized with method *y*.

To investigate whether reductions in time and resource costs are possible for large-scale fits, we make use of QCEngine to investigate the use of semiempirical and machine learning methods for reference data generation. Fitting directly to scans performed using the semiempirical GFN2-xTB (row 2) leads to modest improvements in accuracy from 1.1 to 0.8 kcal/mol RMSE, also with some improvement in the molecular geometries (still measured relative to the default QC scans).

Furthermore, rows 3 and 4 in Table 3 correspond to reference data generated using optimized geometries obtained with GFN2-xTB, combined with single-point energies used to refine the potential energy surface, using either ANI2x or QM at the B3LYP-D3BJ/DZVP level, respectively. We can see that accuracy gains are possible using this flexible, hybrid approach for reference data generation. In particular, for the B3LYP-D3BJ/DZVP//GFN2-xTB method, the RMSE is significantly reduced from 1.1 to 0.6 kcal/mol, with a total computational cost at a fraction of the full QM torsion drive (relative computational costs of all of these methods have been reported elsewhere^49^). All of the BespokeFit augmented FFs tested also show a decrease in the maximum RMSD between the QM reference and MM optimized geometries, despite only a small proportion of the total valence terms of each molecule being optimized. Similar conclusions are reached if we instead focus the analysis on metrics assessing the geometries and relative energies of low-lying minima (Supporting Information Section S2).^50^ Thus, the methods shown in Table 3 represent a hierarchy of increasing accuracy as the reference data generation method becomes more expensive. Users are able to optimize the balance between fitting time and accuracy to suit their needs all through a common interface between BespokeFit and QCEngine.

Having established the accuracy of BespokeFit torsion parameters in gas phase scans, we now move on to computing (as a proof-of-principle) the relative binding free energies of the TYK2 series using the base Parsley FF, and two of our bespoke augmented FFs, namely, BespokeFit (GFN2-xTB) and BespokeFit (B3LYP-D3BJ/DZVP) from Table 3. This protein target was chosen as a system for which conventional FFs tend to perform well,^10^ and so sampling issues are unlikely to affect the interpretation of the data. The fragment FFs were first combined into a single FF, which can be used to parametrize all molecules in the set using the FF combiner CLI tool of BespokeFit. Relative binding free energies were calculated using a workflow based on pmx,^42,51^ which is described in Supporting Information Section S1.3.

**Figure 6:**
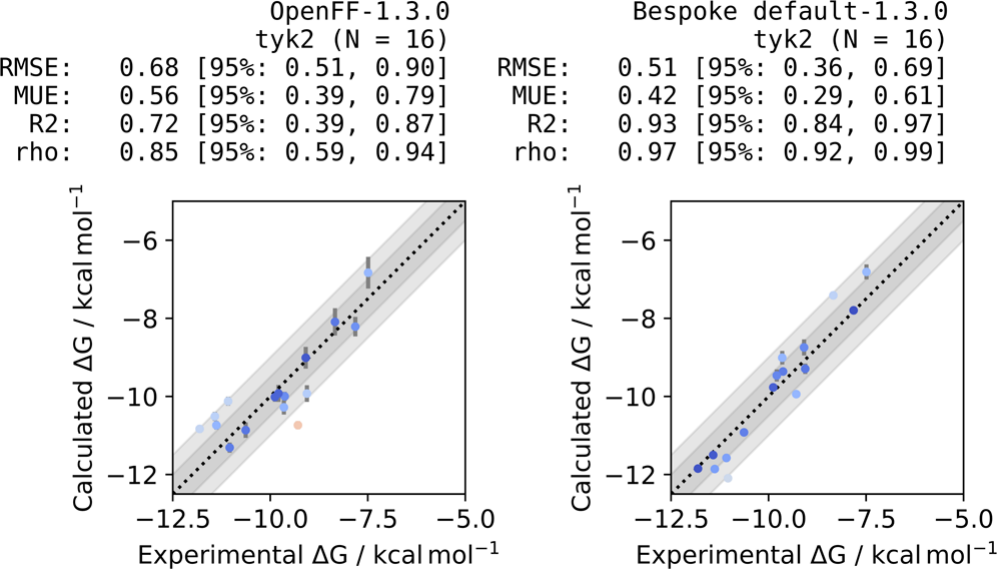
Bespoke dihedral parameters derived with BespokeFit improve the accuracy of binding free energy calculations. Correlation between computed binding free energies and experiment for a congeneric series of TYK2 inhibitors. (Left) Using the base OpenFF Parsley (1.3.0) FF and (right) augmented with bespoke torsion parameters fit to QC data calculated at the B3LYP-D3BJ/DZVP level. Computed results are shifted to have the same mean as the experimental data. Guidelines to aid the eye representing errors of 0.5 and 1 kcal/mol are shown as the dark and light gray-shaded regions, respectively.

Correlations between computed and experimental binding free energies for the base FF (Parsley) and BespokeFit (B3LYP-D3BJ/DZVP) are shown in Figure 6 along with statistics and 95% confidence intervals as reported using cinnabar (formerly Arsenic).^52^ Reassuringly the base FF, Parsley 1.3.0, performs very competitively achieving the sub 1 kcal/mol accuracy required to efficiently guide a drug discovery campaign. But the BespokeFit variant of the FF further improves all of the reported statistical measures. In particular, the correlation between the calculated and experimental binding free energy is improved from 0.72_0.35_^0.87^ to 0.93_0.84_^0.97^ and the confidence interval is significantly narrowed. To further investigate possible reasons for the improvement in accuracy, correlations between the experimental and computed relative binding free energies (ΔΔ*G*) for each of the simulated perturbations are also shown in Figure S6. From these plots, we identify three perturbations (involving five molecules) for the base FF with errors greater than 1 kcal/mol, compared to just one perturbation with the BespokeFit FF. The dihedral potential energy surface scans, before and after bespoke parameter fitting, for the molecules with high errors are plotted in Figure S7. In one case, the base Parsley FF already performs well, but significant improvements in the reproduction of the QM potential energy surfaces are seen in the remaining cases. Together, these data indicate that improvements in dihedral parametrization can translate into improvements in calculated binding free energies, and also show that our choice of fragmentation scheme has generated torsion parameters that transfer well from the fragment to the parent, without introducing any irregularities into the FFs.

As a further experiment, we have also rerun the free energy calculations using the BespokeFit FF fit to xTB torsion scans. Interestingly, as we saw in Table 3, the BespokeFit (GFN2-xTB) FF is intermediate in accuracy, on all measures, between the base FF and the FF fit to the default QC data. For example, the RMS error in binding free energies is 0.64_0.39_^0.93^ kcal/mol (Figure S8). Whether this accuracy hierarchy holds more generally, however, will require further protein–ligand free energy benchmarking. Here, we simply present this case as an example application to protein–ligand binding to show that bespoke torsion parameter fits can be relevant to the accuracy of binding predictions.

## 4 Discussion and Conclusions

Bespoke FF parametrization has the potential to significantly improve the accuracy of binding free energy calculations in drug discovery applications. With increased computing power, resources for data storage and curation, and access to a wide range of high-quality models for generating reference data, prospects for regular high-throughput bespoke FF parametrization are improving. Towards this goal, we present here the open-source OpenFF BespokeFit and OpenFF QCSubmit software packages, which enable bespoke parametrization for SMIRNOFF-based FFs at scale.

We have demonstrated the scalable nature of the combined workflow by optimizing torsion parameters for a diverse set of 199 druglike molecules, which correspond to 671 unique torsion drives for 490 fragments of the input molecules. Keeping track of such datasets would not be feasible without the use of QCSubmit to submit, curate, and retrieve the reference data from QCArchive. The combination of OpenFF BespokeFit, QCSubmit, and QCArchive provides a unique opportunity to reuse QM reference data when deriving bespoke torsion parameters. For example, for a set of 2083 diverse, unseen compounds taken from a recent high-throughput screening campaign,^53^ we find that 4% of the reference torsion drives that would be required to parametrize the dihedral parameters are already present in QCArchive (Supporting Information Section S3). While this currently represents a small, but not insignificant, proportion of the required data, coverage will certainly increase as additional datasets are contributed. In a drug discovery setting, this overlap could be dramatically increased with the careful design of a common torsion dataset composed of molecules from existing project libraries, which is now trivial to build using OpenFF QCSubmit. All datasets used in this study have been serialized to file JSON as an illustration of the reproducibility of the fitting procedure (Supporting Information Section S1.1).

The optimized torsion parameters improve the agreement between our QM reference and MM modeled potential energy surfaces, from 1.1 kcal/mol for the base FF to 0.4 kcal/mol using the default BespokeFit workflow settings. As well as providing a concise base FF, the use of SMIRKS patterns to encode the bespoke torsion terms also means that they are transferable without modification between fragmented molecules and their parents. We have made extensive use of this feature to build bespoke FFs for a congeneric series of inhibitors of TYK2. We find that, while the base Parsley FF (OpenFF 1.3.0) provides competitive accuracy, our BespokeFit FF leads to improvements in all reported free energy statistical measures.

Furthermore, the flexibility of BespokeFit and its interface with QCEngine allows us to investigate the balance between accuracy and speed for a range of QC, semiempirical, and machine learning-based reference data generation methods. Further work will be required to investigate the optimal combinations of methods to generate and refine the reference potential energy surfaces,^4,54,55^ and to determine whether improvements in reference data always lead to improved binding free energy estimates, as we saw here. It seems likely that in some cases sampling, rather than force field quality, may be a limiting factor.

Despite the clear increase in accuracy over the base FF (Table 2 and Figure 6), there is still room for improvement in the reproduction of the underlying reference potential energy surfaces. The infrastructure described here will provide a useful resource for experimenting with improved functional forms, such as coupling between valence terms, improved nonbonded models, and 1-4 scaling interactions. We have not investigated here whether improvements in 1D torsion scans always translate to higher-dimensional scans in cases where there is coupling between neighboring dihedral angles. In such cases, it may be required to allow for fitting to reference data from two-dimensional (2D) torsion drives. Finally, the current approach is quite conservative, as the torsion parameters of every rotatable bond are subject to optimization regardless of the initial accuracy of the base FF. Methods to predict the confidence in torsion parameter accuracy may be helpful in determining which angles would benefit from bespoke parametrization to further increase the throughput and efficiency of the workflow.

All of the software and data used in the current study are freely available and permissively licensed, and a subset of this BespokeFit workflow focusing on bespoke torsions has also been implemented within the Cresset Flare software.^56^ Since the release of the BespokeFit software, we have been active in responding to user issues and we continue to welcome suggestions from the community for future improvements.

## Notes

BespokeFit and QCSubmit are both fully open-source and available under the MIT license on GitHub. Both packages are readily installable using the conda command conda install -c conda-forge openff-bespokefit openff-qcsubmit. Documentation, and examples are available for both packages and can be found following the links on their respective GitHub pages https://github.com/openforcefield/openff-bespokefit and https://github.com/openforcefield/openff-qcsubmit. To provide feedback on the performance of the OpenFF force fields, we highly recommend using the issue tracker at http://github.com/openforcefield/openforcefields. For toolkit feedback, use http://github.com/openforcefield/openff-toolkit. Alternatively, inquiries may be e-mailed to mailto:support@openforcefield.org, though responses to e-mails sent to this address may be delayed and GitHub issues receive higher priority. For information on getting started with OpenFF, see the documentation linked at http://github.com/openforcefield/openforcefield, and note the availability of several introductory examples. Supporting data, including serialized BespokeFit workflow (JSON), combined bespoke force field (XML) for case study 1, notebooks to reproduce raw data for Tables 2 and 3 (IPYNB), notebook to reproduce Figure 5 (IPYNB), force fields and input files for pmx calculations, and raw data (CSV) for Figures 6 and S8, are available at https://doi.org/10.5281/zenodo.7062336.

## Supporting Information Available

Supporting computational methods, example TorsionDrive dataset factory, additional torsion scans, list of TYK2 inhibitors, analysis of free energy outliers, and additional BespokeFit (GFN2-xTB) binding free energies (PDF)
