# Supplementary material for: Open Force Field BespokeFit: Automating Bespoke Torsion Parametrization at Scale
Source: J Chem Inf Model. 2022 Nov 9;62(22):5622–33. doi: 10.1021/acs.jcim.2c01153 (PMC9709916; doi:10.1021/acs.jcim.2c01153)
Supplement: Supplementary file 1 — ci2c01153_si_001.pdf [file ci2c01153_si_001.pdf]

Supporting information for: Open Force Field BespokeFit: Automating Bespoke Torsion Parametrization At Scale

Joshua T. Horton[1], **Simon Boothroyd**[2], **Jeffrey Wagner**[3], **Joshua A. Mitchell**[3], **Trevor Gokey**[4], **David L. Dotson**[3], **Pavan Kumar Behara**[5], **Venkata Krishnan Ramaswamy**[6], **Mark Mackey**[6], **John D. Chodera**[7], **Jamshed Anwar**[8], **David L. Mobley**[4, 5], **Daniel J. Cole**[1,*]

[1]School of Natural and Environmental Sciences, Newcastle University, Newcastle upon Tyne NE1 7RU, United Kingdom.; [2]Boothroyd Scientific Consulting Ltd., 71-75 Shelton Street, London, Greater London, United Kingdom, WC2H 9JQ; [3]The Open Force Field Initiative, Open Molecular Software Foundation, Davis, California 95616, United States; [4]Department of Chemistry, University of California, Irvine, CA 92697, USA; [5]Department of Pharmaceutical Sciences, University of California, Irvine, CA 92697, USA; [6]Cresset, New Cambridge House, Bassingbourn Road, Litlington SG8 0SS, Cambridgeshire, U.K.; [7]Computational & Systems Biology Program, Sloan Kettering Institute, Memorial Sloan Kettering Cancer Center, New York, NY, USA 10065; [8]Department of Chemistry, Lancaster University, Lancaster LA1 4YW, United Kingdom

***For correspondence:**
daniel.cole@ncl.ac.uk (DJC)

S1 Supporting Computational Methods

S1.1 Reference Data Generation

All QM and semi-empirical torsiondrive reference data calculations were performed using the public QCFractal instance, known as QCArchive [1], and are freely available in the torsiondrive dataset "OpenFF-benchmark-ligand-fragments-v2.0". TorsionDrive version 1.1.0 was used to schedule the constrained geometry optimizations which were executed using the geomeTRIC optimization driver version 0.9.7.2 via the QCEngine interface. The QM calculations are stored under the "default" specification which uses the standard QC method and basis B3LYP-D3BJ/DZVP [2–5] used to develop the main line OpenFF FFs. All QM calculations were computed using Psi4 QC package version 1.4 [6]. All semi-empirical calculations are available under the "gfn2xtb" specification under the same dataset name, the calculations were computed using the GFN2-xTB [7] model using xTB version 6.4.1. The scripts used to generate and submit the dataset are available at https://github.com/openforcefield/qca-dataset-submission under the name "2021-08-10-OpenFF-JACS-Fragments-v2.0". The dataset was generated with version 0.2.3 of OpenFF-QCSubmit and version 0.10.0 of the OpenFF-Toolkit. Molecules were fragmented using version 0.1.2 of OpenFF-Fragmenter using both Ambertools version 21.0 and OpenEye version 2021.1.1 backends to perform the AM1 WBO calculations, in order to account for slight differences in the fragments produced with these dependencies. When Ambertools is used to generate the charges, conformers were generated using RDKit version 2021.03.4. All bespoke parameter optimizations were carried out with ForceBalance version 1.9.3 and OpenFF-BespokeFit version 0.1.1 with default BespokeFit settings.

S1.2 Altering the ForceBalance Objective Function

In Case Study 1, we attempted to remove the need for adding a weak (1 kcal/mol/Å^2) restraint to atoms during the MM optimization stage of the fitting procedure. Specifically, we add the difference in internal coordinates of all dihedral angles, measured between each QM and MM optimized structure, into the ForceBalance objective function. The extra term is weighted using the same attenuation method as the energy contributions, meaning that higher energy deviations are down-weighted in favour of low energy conformations (see Section 2.6). The total torsion contribution to the objective is then:

$$L_{torsion}(\Phi) = \frac{1}{{S_f}^2} \frac{\sum_{i=1}^{grid points} w(E_{\mathrm{QM}}(\boldsymbol{x}_i)) \left((E_{\mathrm{QM}}(\boldsymbol{x}_i) - E_{\mathrm{MM}}(\boldsymbol{x}_i, \Phi))^2 + \left(\frac{IC(\boldsymbol{x}_i^{\mathrm{MM}}) - IC(\boldsymbol{x}_i^{\mathrm{QM}})}{d_i} \right)^2 \right)}{\sum_{i=1}^{grid points} w(E_{\mathrm{QM}}(\boldsymbol{x}_i))} \tag{1}$$

where d_i is the dihedral relative scaling factor of $50°$/kcal/mol and $IC(\boldsymbol{x}_i^{\mathrm{MM}})$ and $IC(\boldsymbol{x}_i^{\mathrm{QM}})$ are the dihedral angles in internal coordinates at the MM and QM optimized geometries respectively.

S1.3 Protein-Ligand Binding Free Energies

Protein-ligand binding free energies were computed for the TYK2 complex, from a commonly-used literature benchmark dataset [8]. To aid fair comparison with previously reported results across multiple FFs, including OpenFF 1.3 (Parsley) [9], we have prepared the systems using the same initial coordinates as previous work, which are gathered in the protein-ligand-benchmark repository along with the experimental reference data [10]. Initial conformations were taken from version 0.2.0 of the protein-ligand-benchmark repository. In line with the previous benchmark involving OpenFF FFs we have used AMBER ff99sb*ILDN [11, 12] as the protein FF, and software and simulation settings described in full elsewhere [9]. Three ligand force fields were used, the first parametrized with Parsley-1.3.0 using the OpenFF-Toolkit version 0.10.2, the second augmented with bespoke torsion parameters fit to QM scans, and the last augmented with bespoke torsion parameters fit to semi-empirical xTB scans. A FF suitable for the entire congeneric series was constructed by combining each of the individual molecule-specific FFs via a convenience tool provided as part of the BespokeFit CLI. GROMACS compatible topology files were then created using parmed [13] version 3.4.3, and free energies were estimated using the maximum likelihood estimator [14] based on the Crooks fluctuation theorem [15] applied to non-equilibrium work distributions calculated using a workflow implemented via pmx [16, 17] version 2.0+60.g9a653ed. Non-equilibrium simulations were run using GROMACS

version 2021.5. A total of 60 ns of simulation data was collected for each perturbation in both the solution and bound states. This consisted of a 10 ps NVT equilibration simulation at 298 K, followed by an NPT equilibrium production simulation at 298 K and 1 bar for 6 ns. From this trajectory, 80 snapshots were extracted and used to seed short non-equilibrium simulations for 50 ps in which the ligands were alchemically transformed. This protocol was run in forward and reverse directions, and repeated in triplicate. The free energy statistics and plots were calculated using cinnabar (formerly Arsenic) [10] version0.2.1+1.gb537cdb, which implements best practices for the consistent reporting of binding free energy calculations. All statistical measures are reported with 95% confidence intervals which are obtained with bootstrapping with replacement for 1000 iterations.

Table S1. The performance of Parsley and BespokeFit parameters on the TYK2 dataset, relative to the default QC scans, for the reproduction of local minima in the torsional potential energy surface. Methods in parentheses indicate the reference data used for fitting, where the notation 'x//y' indicates that single point calculations were performed with method x, using geometries optimized with method y.

Force Field	MAE ΔE / kcal/mol	% local minima with $\Delta\theta \leq 20°$ and $\Delta E \leq 1$ kcal/mol
OpenFF 1.3.0	$1.628^{1.803}_{1.469}$	$63.5^{72.1}_{54.8}$
BespokeFit (GFN2-xTB)	$1.003^{1.108}_{0.901}$	$66.3^{76.0}_{56.7}$
BespokeFit (ANI2x//GFN2-xTB)	$0.792^{0.873}_{0.710}$	$74.0^{82.7}_{65.4}$
BespokeFit (B3LYP-D3BJ/DZVP//GFN2-xTB)	$0.536^{0.600}_{0.478}$	$90.4^{96.2}_{84.6}$
BespokeFit (B3LYP-D3BJ/DZVP)	$0.323^{0.363}_{0.285}$	$88.5^{94.2}_{81.7}$

S2 Local Minima Analysis

The ability of a force field to reproduce the positions and relative energies of thermally accessible low-lying energy minima on the QM potential energy surface affects the accuracy of binding free energy calculations. To further investigate the accuracy of the force fields presented in Case Study 2, in this regard, we have implemented the analysis protocol reported as part of the TorsionNet benchmark [18]. In particular, for each low-lying QM local minimum (within 4.2 kcal/mol of the QM global minimum) in the 1D torsion drives, we computed the difference in energy, ΔE, to the nearest MM local minimum. To assess the geometry accuracy, we also recorded the corresponding difference in dihedral angle between each pair of minima, $\Delta\theta$. Table S1 reports the mean absolute error (MAE) in ΔE across all torsion profiles and shows a similar trend to the RMSE reported in Table 3 of the main text. We also report the percentage of the 104 low-lying minima that are recovered by the force field within $20°$ of the QM minimum, and have a relative energy error (between the QM reference and MM value) within 1 kcal/mol. Again, the conclusions are very similar to the RMSD metric reported in Table 3, namely more accurate reference data produce geometries closer to the QC reference. The force field fit to the B3LYP-D3BJ/DZVP data is the most accurate, with the lowest MAE of 0.3 kcal/mol and 88% of local minima recovered.

S3 QCArchive Data Reuse

To illustrate the unique ability of OpenFF BespokeFit to reuse QM reference data stored in the public QCArchive instance, we investigated the number of calculations that could be reused when deriving bespoke torsion parameters for a previously unseen and diverse series of drug-like molecules. The set of 2083 unique molecules, which form part of a high throughput screening library, were taken from an upcoming SAMPL9 challenge [19]. The molecules were fragmented with OpenFF QCSubmit using the same default settings as OpenFF BespokeFit, so as to replicate the process of passing the molecules through the default fitting workflow. This produced 1861 unique molecule fragments and 2929 associated torsion drive scans, which would be required by BespokeFit to derive bespoke torsion parameters for the input molecules. We then used OpenFF QCSubmit to filter QCArchive to identify torsion drives which could be reused, that is scans that are calculated at our target QM method (B3LYP-D3BJ/DZVP) and rotate about the same central bond in a given fragment. Overall, we find 130 compatible scans corresponding to 4% of the total required torsion drives. Considering that the compositions of the existing datasets aim to provide reference data for general, transferable force field optimization, not highly bespoke molecule-specific torsion parameters, the coverage is reasonable and certain to grow over time.

```python
from openff.qcsubmit.factories import TorsiondriveDatasetFactory
from openff.qcsubmit.common_structures import QCSpec
from openff.qcsubmit import workflow_components

# Create a torsiondrive factory which will create QCFractal datasets
factory = TorsiondriveDatasetFactory(
    # Add two specifications to compute the torsiondrives with, including
    # the default OpenFF QM and a custom ANI2x ML specification
    qc_specifications={
        "default": QCSpec(),
        "ani2x": QCSpec(
            program="torchani",
            method="ani2x",
            basis=None,
            spec_name="ani2x",
            spec_description="ANI2x standard specification"
        )
    }
)

# Configure and add workflow components to process the input molecules
# Add a list of allowed elements using symbols or atomic numbers
el_filter = workflow_components.ElementFilter(allowed_elements=[1,6,7,8])
# Fragment the molecules to reduce computational cost and select rotatable bonds
fragmentation = workflow_components.WBOFragmenter(keep_non_rotor_ring_substituents=True)
# Filter molecules which are too big
weight_filter = workflow_components.MolecularWeightFilter()
# Filter molecules with too many rotatable bonds
rotor_filter = workflow_components.RotorFilter(maximum_rotors=3)
# Generate a selection of conformers to seed the torsiondrives
conf_gen = workflow_components.StandardConformerGenerator(max_conformers=4)
# Add the components to the workflow in the order they should be executed
factory.add_workflow_components(el_filter, fragmentation, weight_filter, rotor_filter, conf_gen)
```

Figure S1. A code example showing the construction of a TorsionDrive dataset factory using OpenFF QCSubmit. TorsionDrive datasets following this specification are then made by passing a selection of molecules through the factory.

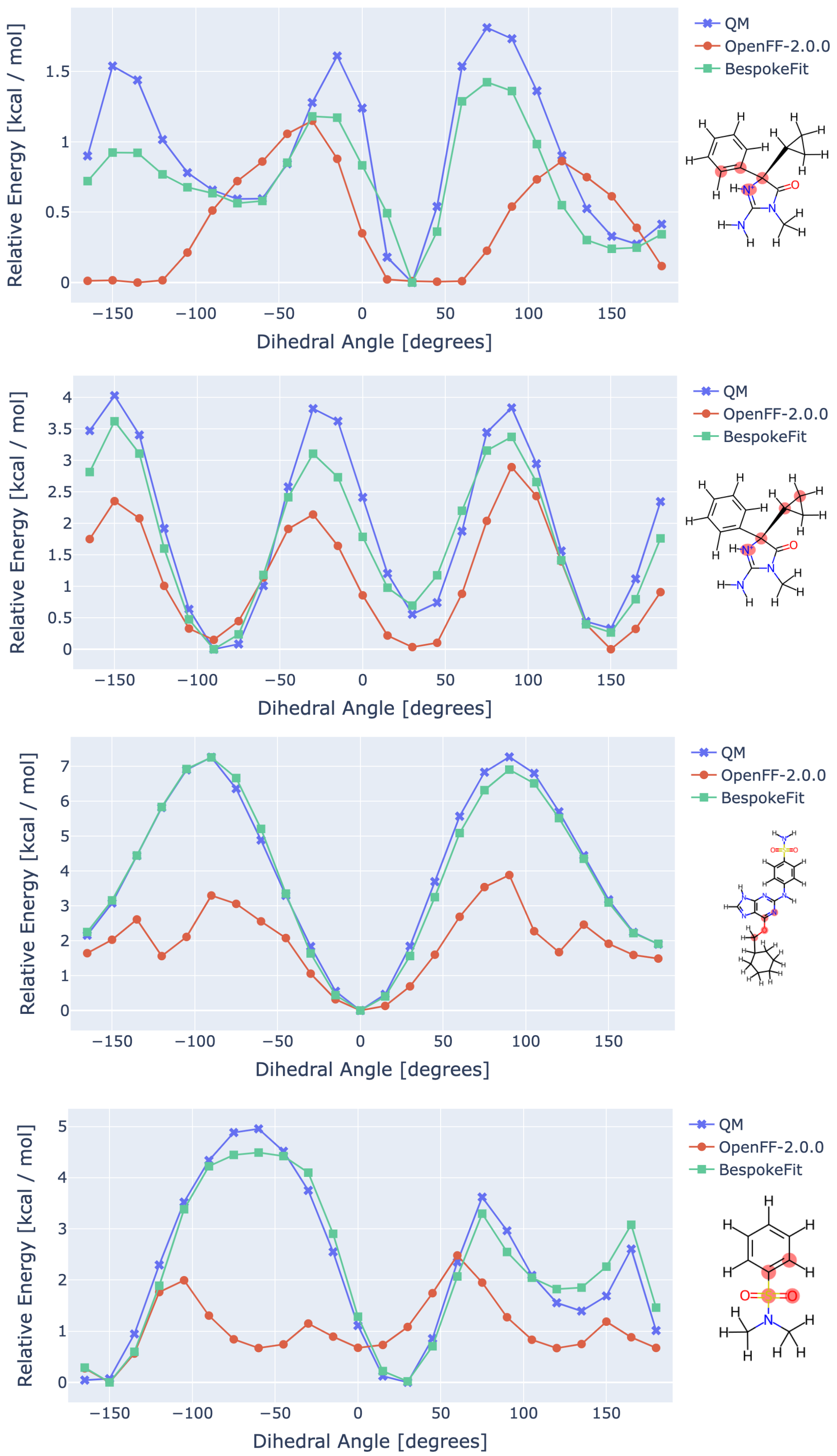

Figure S2. BespokeFit derived torsion parameters improve the agreement between the reference QM and MM potential energy surfaces even in challenging cases. A hand-picked selection of torsion drives is shown for fragments of the Wang benchmark dataset [8]. OpenFF 2.0.0, and its BespokeFit augmented counterpart, are compared to the QM reference data.

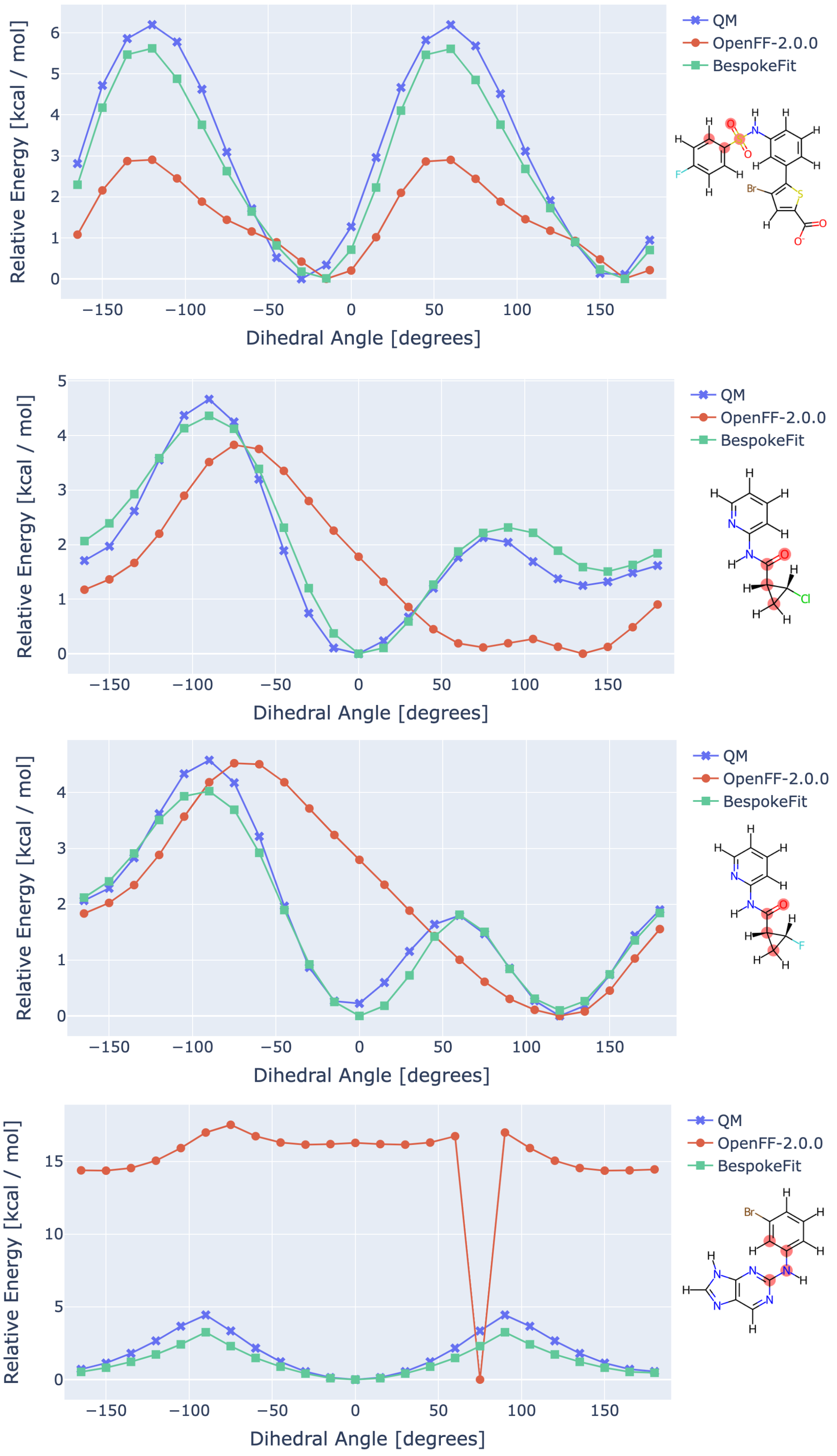

Figure S3. BespokeFit derived torsion parameters improve the agreement between the reference QM and MM potential energy surfaces even in challenging cases. A hand-picked selection of torsion drives is shown for fragments of the Wang benchmark dataset [8]. OpenFF 2.0.0, and its BespokeFit augmented counterpart, are compared to the QM reference data.

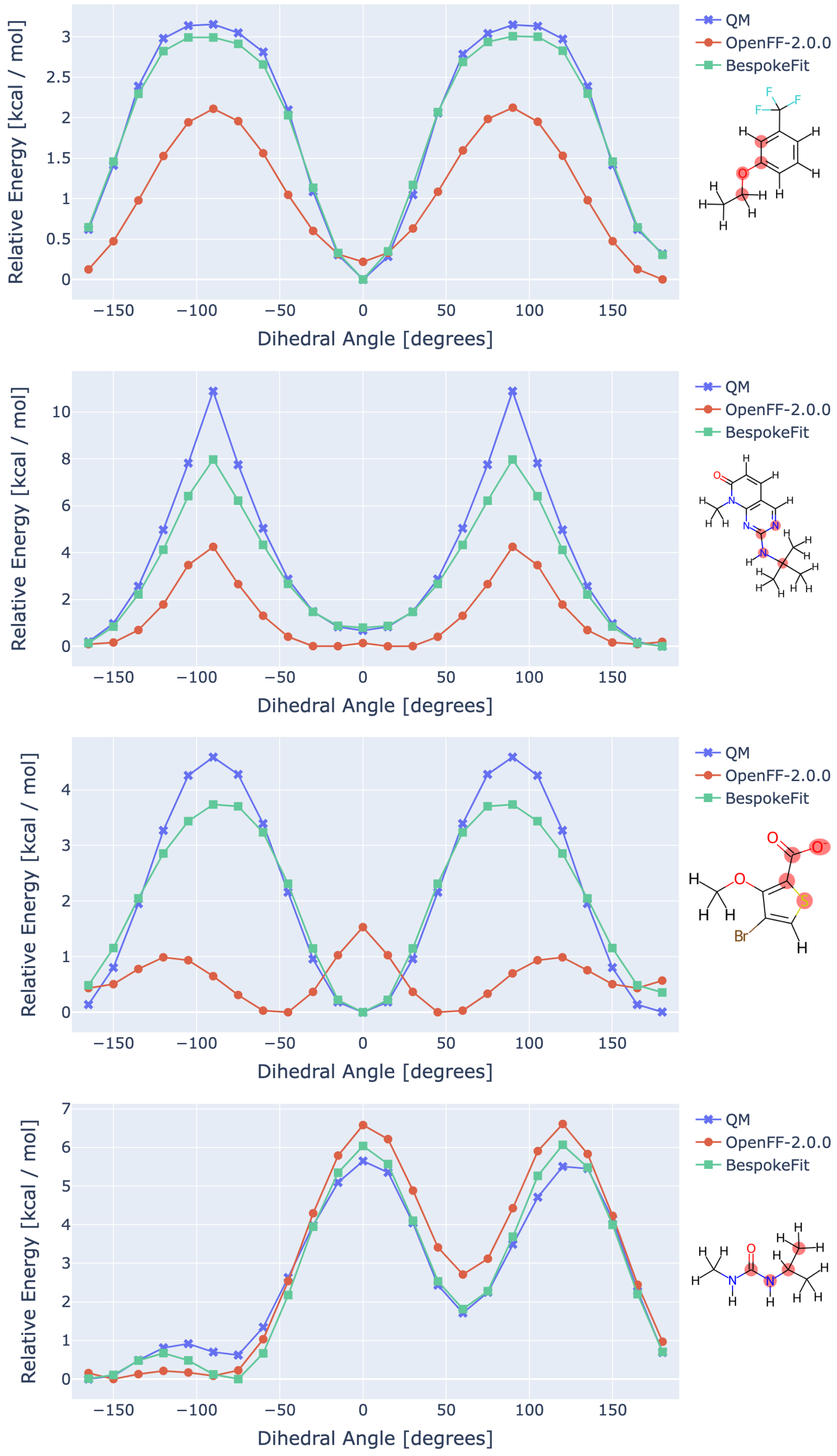

Figure S4. BespokeFit derived torsion parameters improve the agreement between the reference QM and MM potential energy surfaces even in challenging cases. A hand-picked selection of torsion drives is shown for fragments of the Wang benchmark dataset [8]. OpenFF 2.0.0, and its BespokeFit augmented counterpart, are compared to the QM reference data.

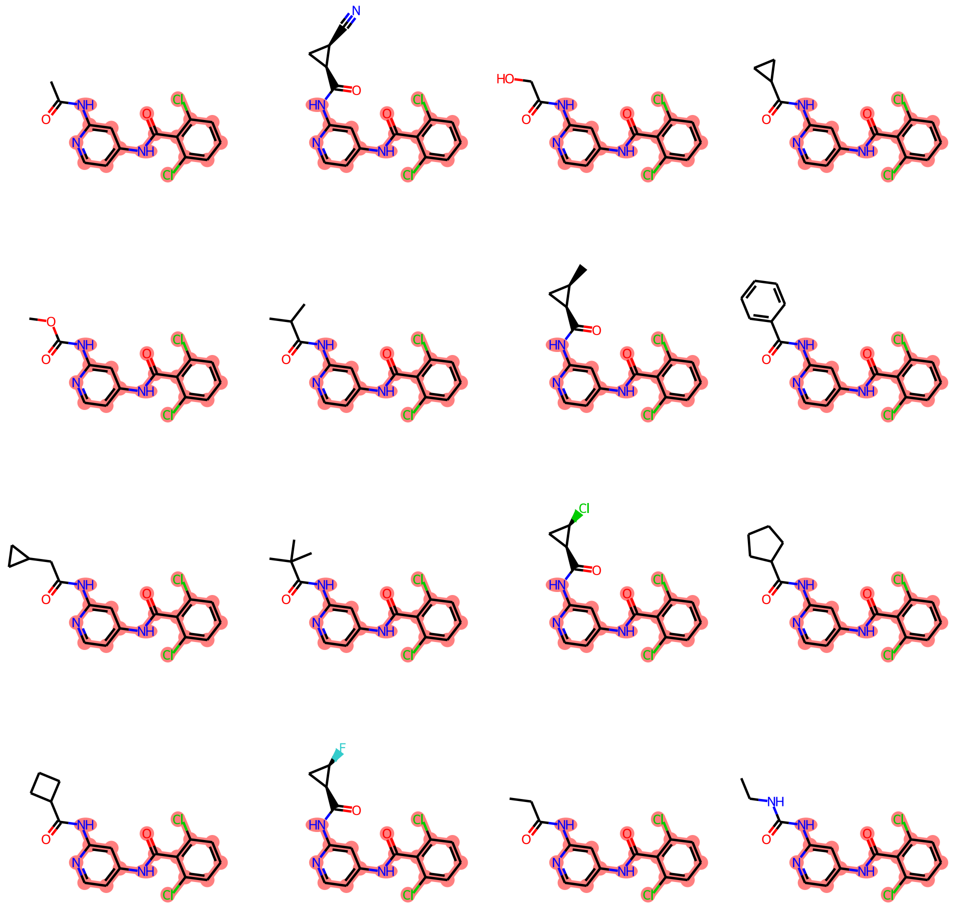

Figure S5. The default fragmentation method in BespokeFit identifies a common core shared between a congeneric series of ligands. Ligands from the TYK2 Wang benchmark system [8] are shown with the common core identified by Fragmenter highlighted in red, hydrogen atoms are removed for clarity.

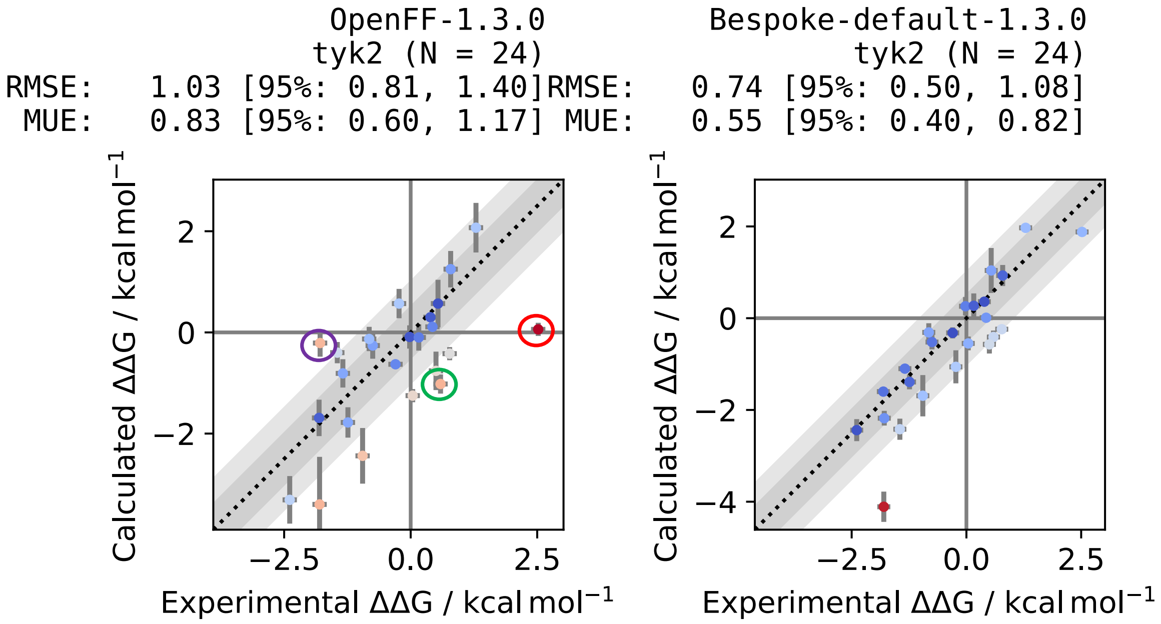

Figure S6. Bespoke dihedral parameters derived with BespokeFit reduce the number of outlier perturbations in binding free energy calculations. Correlation between computed relative binding free energies and experiment for a congeneric series of TYK2 inhibitors. (left) Using the base OpenFF Parsley (1.3.0) FF and (right) augmented with bespoke torsion parameters fit to QC data calculated at the B3LYP-D3BJ/DZVP level. Three outlier perturbations for the base ff have been highlighted in red, green and purple corresponding to the transformations jcm23 → ejm55, ejm42 → ejm55 and ejm31 → ejm46 respectively.

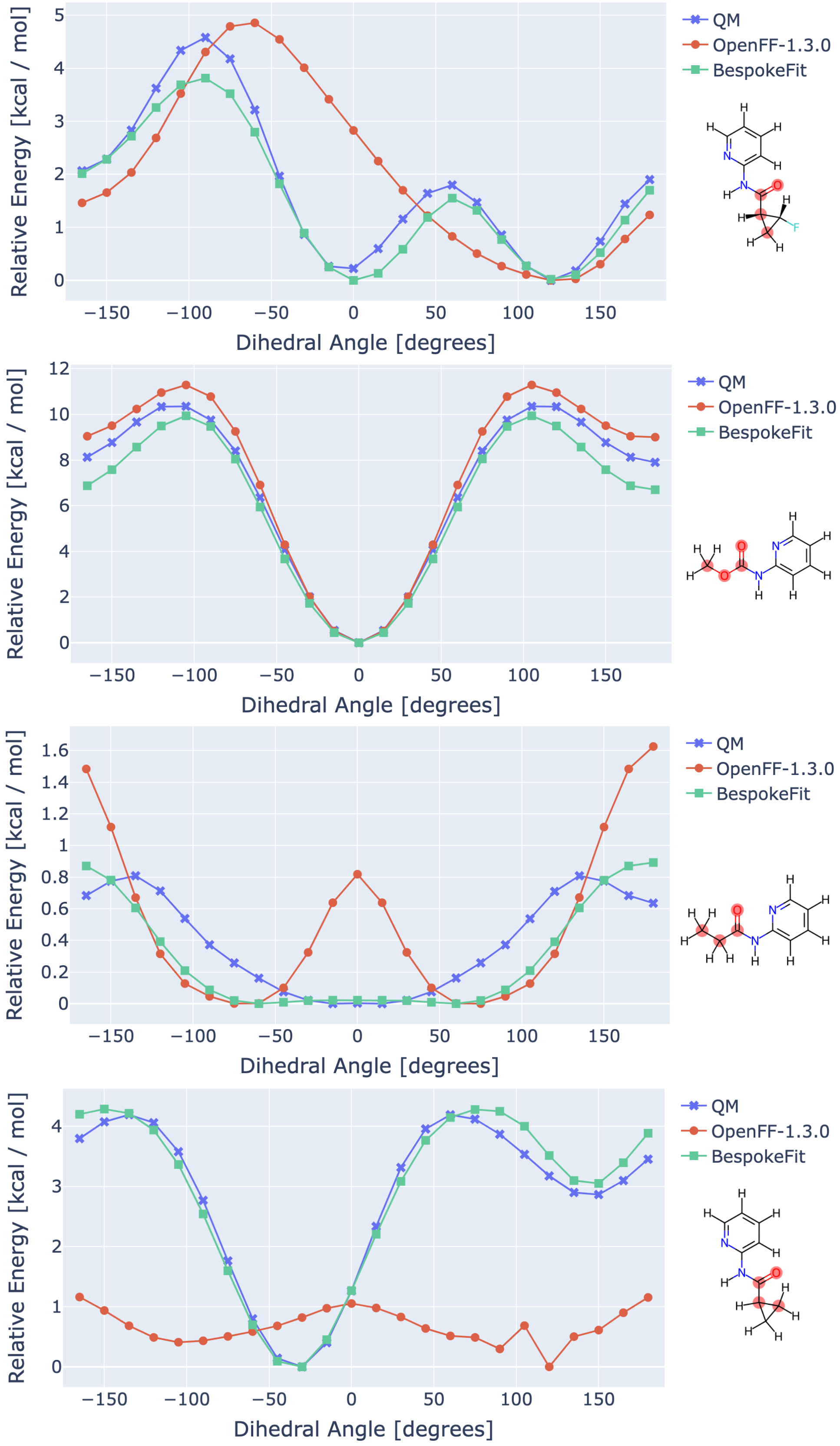

Figure S7. BespokeFit derived torsion parameters improve the agreement between the reference QM and MM potential energy surfaces. Torsion drives are shown for fragments of the outlier molecules from the TYK2 binding free energy benchmark. OpenFF 1.3.0, and its BespokeFit augmented counterpart are compared to the QM reference data for fragments of the ligands: (from the top) 1) jcm23, 2) ejm55, 3) ejm42, 4) ejm46 in the benchmark. Note that ejm31 is excluded due to the terminal group being methyl, which is not scanned as it is assumed to be well modelled by the base FF.

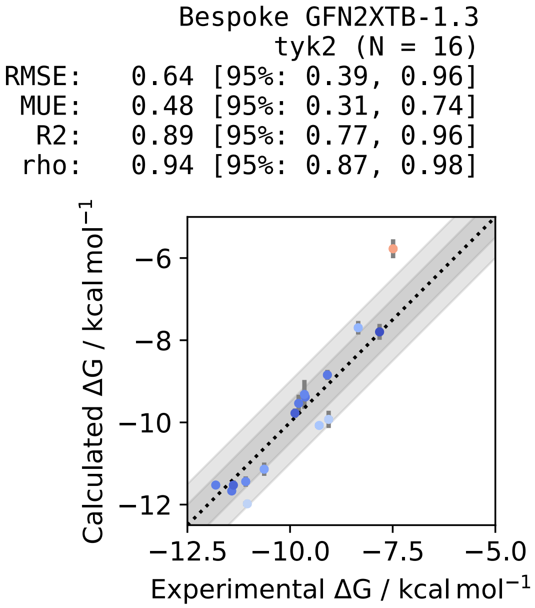

Figure S8. Bespoke dihedral parameters derived with BespokeFit improve the accuracy of binding free energy calculations. Correlation between computed binding free energies and experiment for a congeneric series of TYK2 inhibitors. Using the base OpenFF Parsley (1.3.0) FF augmented with bespoke torsion parameters fit to GFN2-xTB data. Computed results are shifted to have the same mean as the experimental data. Guidelines to aid the eye representing errors of 0.5 and 1 kcal/mol are shown as the dark and light grey shaded regions respectively.

References

[1] **Smith DGA**, Altarawy D, Burns LA, Welborn M, Naden LN, Ward L, Ellis S, Pritchard BP, Crawford TD. The MolSSI QCArchive project: An open-source platform to compute, organize, and share quantum chemistry data. WIREs Computational Molecular Science. 2021; 11(2):e1491. https://wires.onlinelibrary.wiley.com/doi/abs/10.1002/wcms.1491, doi: https://doi.org/10.1002/wcms.1491.

[2] **Becke AD**. Density-functional thermochemistry. III. The role of exact exchange. The Journal of Chemical Physics. 1993; 98(7):5648–5652. doi: 10.1063/1.464913.

[3] **Godbout N**, Salahub DR, Andzelm J, Wimmer E. Optimization of Gaussian-type basis sets for local spin density functional calculations. Part I. Boron through neon, optimization technique and validation. Canadian Journal of Chemistry. 1992; 70(2):560–571. doi: 10.1139/v92-079.

[4] **Grimme S**, Antony J, Ehrlich S, Krieg H. A consistent and accurate ab initio parametrization of density functional dispersion correction (DFT-D) for the 94 elements H-Pu. The Journal of Chemical Physics. 2010; 132(15):154104. doi: 10.1063/1.3382344.

[5] **Grimme S**, Ehrlich S, Goerigk L. Effect of the damping function in dispersion corrected density functional theory. Journal of Computational Chemistry. 2011; 32(7):1456–1465. doi: https://doi.org/10.1002/jcc.21759.

[6] **Smith DGA**, Burns LA, Simmonett AC, Parrish RM, Schieber MC, Galvelis R, Kraus P, Kruse H, Di Remigio R, Alenaizan A, James AM, Lehtola S, Misiewicz JP, Scheurer M, Shaw RA, Schriber JB, Xie Y, Glick ZL, Sirianni DA, O'Brien JS, Waldrop JM, Kumar A, Hohenstein EG, Pritchard BP, Brooks BR, Schaefer HF, Sokolov AY, Patkowski K, DePrince AE, Bozkaya U, King RA, Evangelista FA, Turney JM, Crawford TD, Sherrill CD. PSI4 1.4: Open-source software for high-throughput quantum chemistry. The Journal of Chemical Physics. 2020; 152(18):184108. doi: 10.1063/5.0006002.

[7] **Bannwarth C**, Ehlert S, Grimme S. GFN2-xTB—An Accurate and Broadly Parametrized Self-Consistent Tight-Binding Quantum Chemical Method with Multipole Electrostatics and Density-Dependent Dispersion Contributions. Journal of Chemical Theory and Computation. 2019; 15(3):1652–1671. https://doi.org/10.1021/acs.jctc.8b01176, doi: 10.1021/acs.jctc.8b01176.

[8] **Wang L**, Wu Y, Deng Y, Kim B, Pierce L, Krilov G, Lupyan D, Robinson S, Dahlgren MK, Greenwood J, Romero DL, Masse C, Knight JL, Steinbrecher T, Beuming T, Damm W, Harder E, Sherman W, Brewer M, Wester R, Murcko M, Frye L, Farid R, Lin T, Mobley DL, Jorgensen WL, Berne BJ, Friesner RA, Abel R. Accurate and Reliable Prediction of Relative Ligand Binding Potency in Prospective Drug Discovery by Way of a Modern Free-Energy Calculation Protocol and Force Field. Journal of the American Chemical Society. 2015; 137(7):2695–2703. https://doi.org/10.1021/ja512751q, doi: 10.1021/ja512751q.

[9] **Qiu Y**, Smith DGA, Boothroyd S, Jang H, Hahn DF, Wagner J, Bannan CC, Gokey T, Lim VT, Stern CD, Rizzi A, Tjanaka B, Tresadern G, Lucas X, Shirts MR, Gilson MK, Chodera JD, Bayly CI, Mobley DL, Wang LP. Development and Benchmarking of Open Force Field v1.0.0—the Parsley Small-Molecule Force Field. Journal of Chemical Theory and Computation. 2021; 17(10):6262–6280. https://doi.org/10.1021/acs.jctc.1c00571, doi: 10.1021/acs.jctc.1c00571.

[10] **Hahn DF**, Bayly CI, Macdonald HEB, Chodera JD, Mey AS, Mobley DL, Benito LP, Schindler CE, Tresadern G, Warren GL. Best practices for constructing, preparing, and evaluating protein-ligand binding affinity benchmarks. arXiv preprint arXiv:210506222. 2021; .

[11] **Hornak V**, Abel R, Okur A, Strockbine B, Roitberg A, Simmerling C. Comparison of multiple Amber force fields and development of improved protein backbone parameters. Proteins: Structure, Function, and Bioinformatics. 2006; 65(3):712–725. doi: https://doi.org/10.1002/prot.21123.

[12] **Lindorff-Larsen K**, Piana S, Palmo K, Maragakis P, Klepeis JL, Dror RO, Shaw DE. Improved side-chain torsion potentials for the Amber ff99SB protein force field. Proteins: Structure, Function, and Bioinformatics. 2010; 78(8):1950–1958. doi: https://doi.org/10.1002/prot.22711.

[13] **Shirts MR**, Klein C, Swails JM, Yin J, Gilson MK, Mobley DL, Case DA, Zhong ED. Lessons learned from comparing molecular dynamics engines on the SAMPL5 dataset. Journal of computer-aided molecular design. 2017; 31(1):147–161. doi: 10.1007/s10822-016-9977-1.

[14] **Shirts MR**, Bair E, Hooker G, Pande VS. Equilibrium Free Energies from Nonequilibrium Measurements Using Maximum-Likelihood Methods. Phys Rev Lett. 2003 Oct; 91:140601. doi: 10.1103/PhysRevLett.91.140601.

[15] **Crooks GE**. Entropy production fluctuation theorem and the nonequilibrium work relation for free energy differences. Phys Rev E. 1999 Sep; 60:2721–2726. doi: 10.1103/PhysRevE.60.2721.

[16] **Gapsys V**, Michielssens S, Seeliger D, de Groot BL. pmx: Automated protein structure and topology generation for alchemical perturbations. Journal of Computational Chemistry. 2015; 36(5):348–354. doi: https://doi.org/10.1002/jcc.23804.

[17] **Gapsys V**, Pérez-Benito L, Aldeghi M, Seeliger D, van Vlijmen H, Tresadern G, de Groot BL. Large scale relative protein ligand binding affinities using non-equilibrium alchemy. Chem Sci. 2020; 11:1140–1152. http://dx.doi.org/10.1039/C9SC03754C, doi: 10.1039/C9SC03754C.

[18] **Rai BK**, Sresht V, Yang Q, Unwalla R, Tu M, Mathiowetz AM, Bakken GA. TorsionNet: A Deep Neural Network to Rapidly Predict Small-Molecule Torsional Energy Profiles with the Accuracy of Quantum Mechanics. Journal of Chemical Information and Modeling. 2022; 62(4):785–800. doi: 10.1021/acs.jcim.1c01346, pMID: 35119861.

[19] **Amezcua M**, Mobley DL, samplchallenges/SAMPL9: Version 0.1: Initial files for SAMPL9 host-guest. Zenodo; 2021. https://doi.org/10.5281/zenodo.5485849, doi: 10.5281/zenodo.5485849.
